# A case of hypercalcemia from *Pneumocystis jirovecii* in an immunosuppressed non-HIV patient

**DOI:** 10.1186/s12890-024-03007-8

**Published:** 2024-04-24

**Authors:** Vishrut Gulhati, Janeve Desy, Christina S. Thornton

**Affiliations:** 1 Division of Respiratory Medicine, Department of Medicine, University of Calgary, T2N 4N1 Calgary, AB Canada; 2 Department of Microbiology, Immunology and Infectious Diseases, University of Calgary Cumming School of Medicine, Calgary, AB Canada

**Keywords:** *Pneumocystis jirovecii*, Hypercalcemia, Pulmonary infections

## Abstract

**Background:**

The prevalence of non-HIV related *Pneumocystis jirovecii* pneumonia (PJP) is increasing with use of immunosuppressive therapies. There are case reports of solid organ transplant recipients on immunosuppressive therapy presenting with mild hypercalcemia, leading to a diagnosis of PJP. Recent studies have shown efficacy of PJP prophylaxis for patients treated with rituximab with a favourable adverse effect profile.

**Case Presentation:**

A 78-year-old male with a history of PR3-ANCA vasculitis, chronic kidney disease and heart failure with reduced ejection fraction presented to our tertiary care hospital with a two-week history of confusion and non-productive cough. Background immunosuppression with rituximab was completed every six months. The patient was found to have hypercalcemia and new infiltrates and ground glass opacities on cross-sectional imaging. Bronchoscopy was performed that was positive for *Pneumocystis jirovecii*. He was treated with 21 days of trimethoprim-sulfamethoxazole and prednisone with resolution of symptoms and hypercalcemia.

**Conclusions:**

Herein, we present a novel case of PJP in a non-transplant recipient preceded by hypercalcemia. Our case demonstrates the importance for a high suspicion for PJP in chronically immunosuppressed patients on rituximab presenting with PTH-independent hypercalcemia.

## Introduction

PJP pneumonia is a life-threatening infection mainly among immunocompromised hosts [1]. The prevalence of non-HIV related PJP is increasing with emerging immunosuppressive therapies for chronic illnesses [2]. PJP infection outside of the HIV setting often presents with greater severity (i.e. abrupt respiratory failure) and carries an all-cause mortality of up to 30%, which is more severe compared to PJP infection in patients with HIV [3]. Diagnosis of PJP in this setting may be elusive given the often indolent presentation. Hypercalcemia is not classically associated with PJP infection; however, there have been several case reports in the literature of PJP occurring in kidney and liver transplant recipients [4, 5]. We report a novel case of PJP in a non-transplant recipient, on rituximab, preceded by hypercalcemia in addition to the classic respiratory symptoms. This case addresses a knowledge gap whereby clinicians should consider PJP infections on the differential for patients presenting with hypercalcemia, in the appropriate setting.

## Case report

**Fig. 1:**
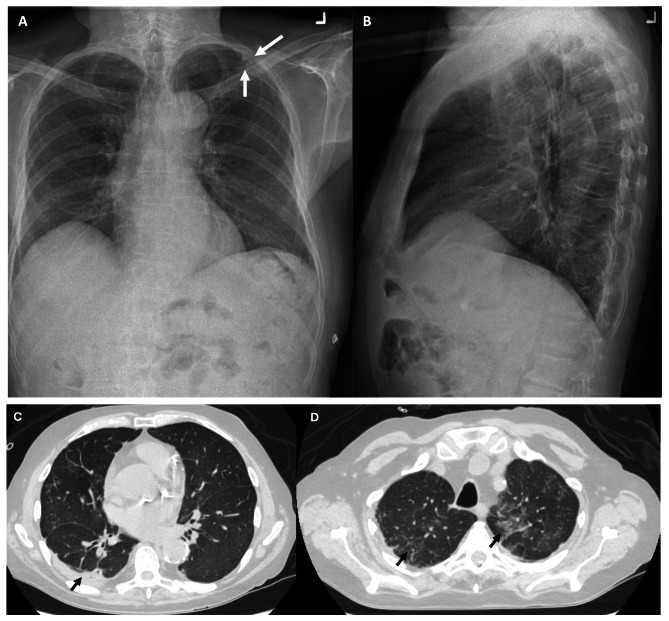
Chest radiography (panel **A**– PA view, panel **B**– lateral view) shows dense pleuroparencymal scarring in the left upper lobe (white arrow) that had been present for the preceding six months. CT chest shows progressive bilateral right more than left peripheral consolidation (black arrows) with distortion (panel **C**). Diffuse bilateral patchy ground glass (black arrows) in the upper lobes were present (panel **D**)

**Fig. 2:**
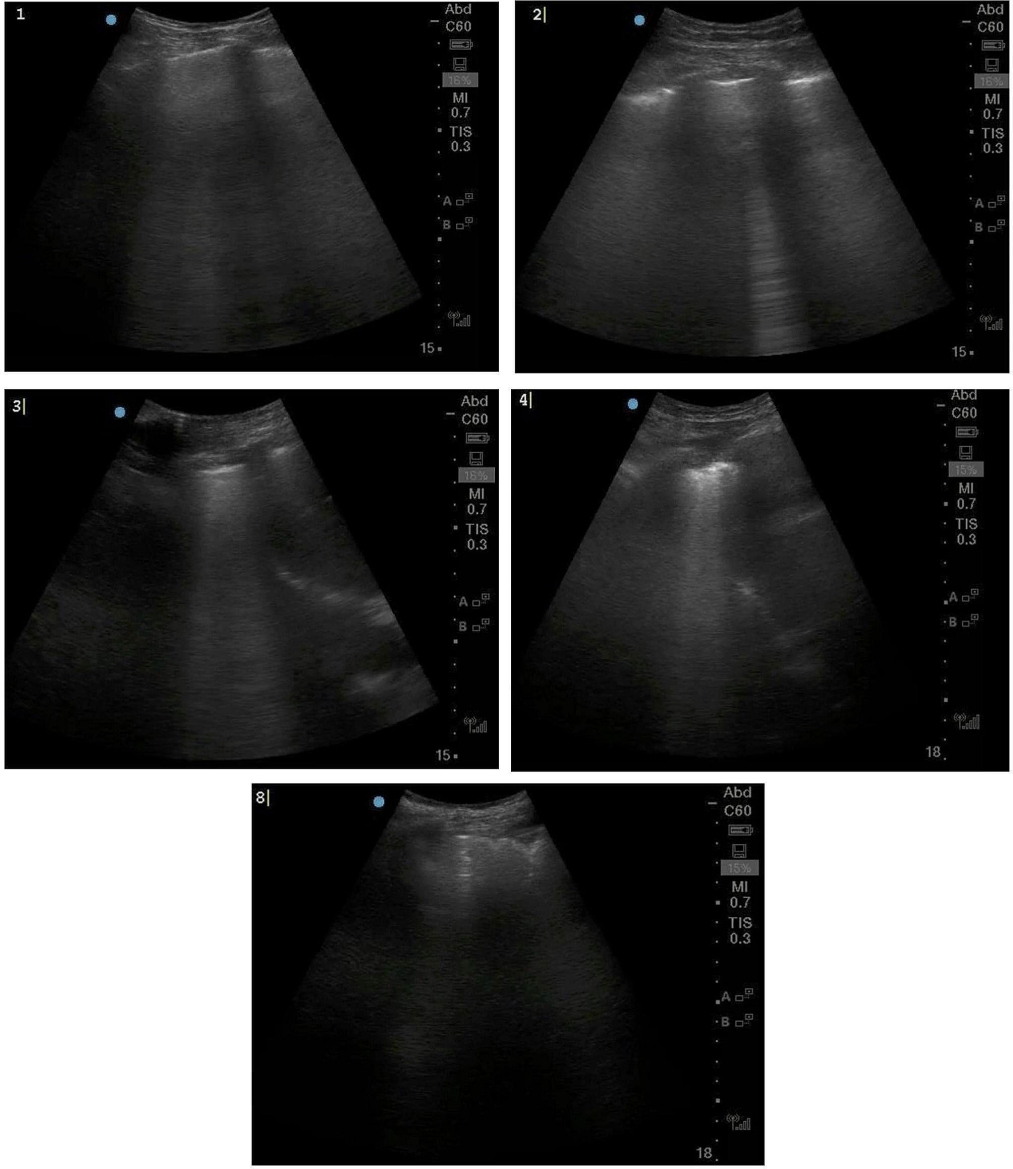
Point of care ultrasound shows B lines in lung zones 1, 2, 3, and 4 some areas of pleural irregularity, and subpleural consolidation in zone 8 (scanned in transverse orientation)

A 78-year-old male with a history of PR3-ANCA vasculitis, chronic kidney disease and heart failure with reduced ejection fraction presented with a two-week history of confusion and non-productive cough. He was maintained on rituximab maintenance for ANCA vasculitis every six months without vitamin D supplementation. He was started on rituximab approximately 13 months prior to initial presentation, having received an induction dose over four weeks for a total of 2000 mg. This included a tapering regimen of prednisone 25 mg daily at time of induction and two maintenance doses of 500 mg of rituximab. During induction with prednisone, PJP prophylaxis was given but was subsequently discontinued with completion of steroids. Physical exam was unremarkable including cardiovascular, pulmonary, and neurologic exams. On presentation, laboratory values revealed an acute kidney injury with creatinine of 356umol/L (eGFR of 13; baseline of 250umol/L, reference range 50-120umol/L). CBC showed hemoglobin 110 g/L (baseline given CKD), platelets 231, leukocytes 7.1 109/L, eosinophils mildly elevated to 0.9 109/L (normalized two days later). Urinalysis showed trace protein and absence of blood. Ionized calcium was elevated at 1.76mmol/L (reference range 1.15-1.35mmol/L) with phosphate 1.84 mmol/L (reference range 0.70-1.50mmol/L). Parathyroid hormone level was low at 8.8ng/L (reference range 15-57ng/L), 1,25-Dihydroxyvitamin D3 (calcitriol) was elevated (299pmol/L, reference range 60-208pmol/L) while 25-hydroxyvitamin D3 (calcidiol) was normal (126nmol/L, reference range 50-200nmol/L). Repeat ANCA panel showed MPO level < 0.2 AI and PR3 2.2 AI (MPO < 0.2 with PR3 12.1 two months prior). Repeat HIV screen remained negative for mixed antigen/antibody detection. Serum and urine protein electrophoresis were negative for monoclonal proteins. Serum free light chains showed a normal kappa/lambda protein ratio of 1.12. Initial chest x-ray (Fig. 1A and B) showed pleuro-parenchymal scarring that was stable compared to an x-ray six months earlier. Point of Care Ultrasound (POCUS) demonstrated B lines in lung zones 1, 2, 3, and 4 with some areas of pleural irregularity, as well as a shred sign, suggestive of consolidation, in zone 8 (Fig. 2) and therefore a high-resolution CT chest was performed which (Fig. 1C and D) showed interval development of dense ground glass opacities.

Given the atypical presentation, the decision was made to perform bronchoscopy. Cultures from bronchoalveolar lavage confirmed diagnosis of PJP. The patient was treated with trimethoprim-sulfamethoxazole (TMP-SMX) at 20 mg/kg three times per day along with prednisone 40 mg twice daily for 21 days. AKI resolved with serum creatinine improving to baseline level of 242 umol/L (eGFR of 21) prior to discharge. Ionized calcium normalized to 1.26 mmol/L prior to discharge. With treatment, hypercalcemia, confusion and respiratory symptoms resolved prior to discharge and has continued to remain stable.

## Discussion

Herein, we present a case where PJP and hypercalcemia has presented with nonspecific clinical symptoms. Our results are in line with other reports of atypical presentations of PJP [6, 7] and emphasizes the importance of recognition in at-risk patients. In our case, our patient was not on empiric PJP prophylaxis given use of primarily rituximab for immunosuppression. While current guidelines suggest TMP-SMX prophylaxis is effective in reducing the incidence of PJP (RR 0.15, 95% CI 0.04–0.62), in trials comparing PJP prophylaxis vs. placebo or no treatment there was no significant effect on all-cause mortality [8]. Taken together, there is a lack of consensus around guideline-based recommendations towards PJP prophylaxis in those immunosuppressed outside of the HIV setting. Recently, in a large-centre retrospective analysis, Park et al. reviewed the efficacy and safety of primary PJP prophylaxis, with TMP-SMX, in patients receiving rituximab [9]. They demonstrated that over nearly 2600 person-years, 92 PJP infections occurred, with a mortality rate of 27%. Notably, patients receiving PJP prophylaxis showed significantly lower incidence of PJP (hazard ratio 0.20, 95% CI 0.10–0.42) and related mortality (hazard ratio 0.21, 95% CI 0.05–0.84]). Intention to treat analysis showed the number needed to treat was 32 and number needed to harm, from a serious adverse drug reaction, was 101. Overall, these findings strongly suggest that potential benefit from TMP-SMX prophylaxis for patients receiving rituximab outweigh any likely harm.

Our case was unique in that one of the presenting features associated with PJP infection was unexplained hypercalcemia. The mechanism for hypercalcemia in PJP is thought to be similar to that of other granulomatous diseases. The presence of inflammatory granulomas rich in pulmonary alveolar macrophages and monocytes, capable of vitamin D activation is felt to be the underlying etiology [10–12]. Pulmonary alveolar macrophages have been shown to produce 1,25(OH)2D, and has been seen in other conditions including sarcoidosis, silicone-related granulomatosis, and infections (i.e. tuberculosis, histoplasmosis, *Bartonella henselae* and fungal infections) [13–19]. In PJP-associated hypercalcemia in transplant patients, similar observations have been seen [7]. Although there is limited evidence of a granulomatous reaction directly linked to PJP, exploring the potential pathophysiological connection between PJP and 1,25(OH)2D may be of future interest—such as through our case which may explain the elevated calcitriol and normal calcidiol level in our patient, despite appropriately low PTH.

This case demonstrates an atypical presentation of PJP infection in a chronically immunosuppressed patient on rituximab, presenting with PTH-independent hypercalcemia. To our knowledge, our case is the first outside of transplant recipients. Recent studies have supported that PJP-prophylaxis should be strongly considered in non-HIV patients receiving rituximab. A high index of suspicion will allow for timely diagnosis and treatment and avoid complications of untreated PJP infection.

## Data Availability

The datasets used and/or analysed during the current study available from the corresponding author on reasonable request.Written informed consent for publication was obtained from the patient and is available for review upon request.

## Abbreviations

PJP: *Pneumocystis jirovecii*
PR3: Proteinase 3
ANCA: Antineutrophil cytoplasmic antibodies
POCUS: Point of care ultrasound
CT: Computed tomography
TMP-SMX: Trimethoprim-sulfamethoxazole
HIV: Human immunodeficiency virus
RR: Relative risk
CI: Confidence interval
PTH: Parathyroid hormone
